# Protective mechanism of fruit vinegar polyphenols against AGEs-induced Caco-2 cell damage

**DOI:** 10.1016/j.fochx.2023.100736

**Published:** 2023-06-05

**Authors:** Qian Wu, Yingfei Kong, Yinggang Liang, Mengyao Niu, Nianjie Feng, Chan Zhang, Yonggang Qi, Zhiqiang Guo, Juan Xiao, Mengzhou Zhou, Yi He, Chao Wang

**Affiliations:** a Key Laboratory of Fermentation Engineering (Ministry of Education), Hubei Key Laboratory of Industrial Microbiology, National “111” Center for Cellular Regulation and Molecular Pharmaceutics, Hubei Research Center of Food Fermentation Engineering and Technology, Hubei University of Technology, Wuhan 430068, Hubei, China; b Beijing Laboratory of Food Quality and Safety, School of Food and Chemical Engineering, Beijing Technology and Business University, Beijing 100048, China; c State Key Laboratory of Marine Resource Utilization in South China Sea/Ministry of Education, Key Laboratory of Food Nutrition and Functional Food of Hainan Province/Engineering Research Center of Utilization of Tropical Polysaccharide Resources/School of Food Science and Engineering, Hainan University, Haikou, China; d National R&D Center for Se-rich Agricultural Products Processing, Hubei Engineering Research Center for Deep Processing of Green Se-rich Agricultural Products, School of Modern Industry for Selenium Science and Engineering, Wuhan Polytechnic University, Wuhan 430023, China

**Keywords:** Fruit vinegar, Polyphenols, Advanced glycation end products, Inhibition, Oxidative stress

## Abstract

Accumulation of advanced glycation end products (AGEs) is linked with development or aggravation of many degenerative processes or disorders. Fruit vinegars are rich in polyphenols that can be a good dietary source of AGEs inhibitors. In this study, eight kinds of vinegars were prepared. Among them, the highest polyphenol and flavonoid content were orange vinegar and kiwi fruit vinegar, respectively. Ferulic acid, vanillic acid, chlorogenic acid, *p*-coumaric acid, caffeic acid, catechin, and epicatechin were main polyphenols in eight fruit vinegars. Then, we measured the inhibitory effect of eight fruit vinegars on fluorescent AGEs, and found that orange vinegar had the highest inhibitory rate. Data here suggested that orange vinegar and its main components catechin, epicatechin, and *p*-coumaric acid could effectively reduce the level of ROS, RAGE, NADPH and inflammatory factors in Caco-2 cells. Our research provided theoretical basis for the application of orange vinegar as AGEs inhibitor.

## Highlights

• Fruit vinegars is a good dietary source of AGEs inhibitors.
• Orange vinegar had the highest AGEs inhibitory rate.
• Orange vinegar can protect against AGEs-induced Caco-2 cell damage.

## 1 Introduction

AGEs were first proposed by Professor Vlassara in 1984 (Vlassara, Brownlee, & Cerami, 1984). They are a group of compounds formed by non-enzymatic glycosylation between ketones or aldehyde groups of sugars and amino groups of proteins. AGEs can be formed endogenous from sugars and proteins at 37 ℃, and may also be formed during food processing. In addition, AGEs are combined with receptor for advanced glycation endproducts (RAGE) on the corresponding cells of the cell surface to promote the production of intracellular reactive oxygen species (ROS). The role of nicotinamide adenine dinucleotide phosphate (NADPH) oxidase and the mitochondrial pathway is to participate in the generation of intracellular ROS (Kellow, & Coughlan, 2015). AGEs caused by reactive oxygen species generation, activate oxidative stress could result in a variety of chronic metabolic diseases, such as diabetic kidney disease, Alzheimer's disease, atherosclerosis and osteoarthritis (Aragno, & Mastrocola, 2017). Therefore, AGEs have long been considered potent toxic molecules that promote host cell death and contribute to organ damage in human. Effective AGEs inhibitors can prevent or delay the formation and accumulation of AGEs, thereby inhibiting the development of disease. Although aminoguanidine (AG) and other inhibitors have a good inhibitory effect on glycosylation, they also produce serious toxic and side effects. Therefore, non-toxic side effects of natural food inhibitors are gradually paid attention to.

Fruit vinegar is a kind of popular drink which has color, flavor and functional properties. The positive correlation between consumption of fruit vinegar rich in nutrients and good health is well known (Zhu et al., 2019, Ho et al., 2017, Wei et al., 2023). Scientific research has reported that different fruit vinegars vary in their phenolic composition and contents, due to different raw materials (Liu, Tang, Zhao, Gan, & Li, 2019). Ren et al. found that the polyphenol in apple cider vinegar was mainly chlorogenic acid, while the highest polyphenol in persimmon vinegar and kiwi vinegar was gallic acid (Ren et al., 2017). Jun-Hui Choi et al. found that the contents of chlorogenic acid, caffeic acid and rutin in Cudrania Tricuspidata fruits vinegar were 331.9 ± 5.9, 46.2 ± 1.3 and 142.9 ± 4.2 μg/mL, respectively (Choi, Kim, Yeo, & Kim, 2020). Studies have shown that polyphenols can inhibit AGEs formation *in vitro* and *in vivo*, but the effect of fruit vinegar on AGEs inhibition is rarely reported (Zhu et al., 2020, Zhang et al., 2021).

Therefore, we brewed eight fruit vinegars to analyze their polyphenols components and further to investigate their protective mechanism against AGEs-induced Caco-2 cell damage.

## 2 Materials and methods

### 2.1 Chemicals

Glucose and bovine serum albumin (BSA) were provided by China National Pharmaceutical Group. Saccharomyces cerevisiae was supplied by Angel Yeast Co., Ltd (Yichang, China). 1,1-diphenyl-2-trinitrophenylhydrazine (DPPH), 2,4,6-tri(2-pyridyl)-1,3,5-trianzine (TPTZ), ferric chloride hexahydrate, trichloroacetic acid, ethanol, and dimethyl sulfoxide were purchased from Sinopharm Chemical. Catechin, epicatechin and *p*-Coumaric acid were bought from Maclean Biotech. Non-essential amino acids and minimum essential medium (MEM) were made available from Hyclone. Fetal bovine serum and sterile PBS were obtained from Gibco. Dimethyl sulfoxide was carried out by Sigma. 0.25% trypsin-EDTA were purchased from Jinuo Biotechnology (Hangzhou, China).

### 2.2 Brewing of fruit vinegar

Eight kinds of ripe and fresh fruits were used, watermelons, grapes, kiwis, apples, pears, oranges, mangos, and persimmons, which were peeled and squeezed in a juicer. After juices were filtered and pasteurized (68 ℃, 30 mins), the sugar content of supernatant was determined using a brix meter and adjusted the sugar content to 15–18° by adding white sugar or deionized water to conducive the growth of microorganisms. Then, 1 g of Angel Yeast mixed with 2% sugar water, and activated at 30 ℃ for half an hour. The juice inoculated with 3% volume of yeast and fermented with a 30 ℃ shaker in an airtight manner (Ubeda et al., 2011). Gas production was assessed by observing the change in the size of the balloon attached to the fermentation flask. Stopped fermentation after the balloon no longer changes. Alcoholic content was measured by using an PT-1 alcoholmeter (Pute company, Beijing, China). *Acetobacillus pasteurii* AS1.41 activated with acetic acid bacteria liquid medium (2% glucose, 2% yeast powder) for three generations, and then inoculated into 200 mL juice at a volume ratio of 3% at 30 ℃ to ferment until the pH no longer changes around 3.5 (15–20 d). The fermented fruit vinegar was centrifuged, filtered, sterilized, and stored at 4 ℃ for use.

### 2.3 Determination of total flavonoid and polyphenols contents in fruit vinegars

#### 2.3.1 Determination of total flavonoid contents

Total flavonoid contents in eight vinegars were determined according to Formagio A.S.N method with some modification (Formagio, Volobuff, Santiago, Cardoso, Vieira, & Valdevina Pereira, 2014). For each analysis, 0.25 mL fruit vinegar and 1.25 mL water mixed with 75 μL of 5% NaNO_2_ solution by turbine mixer (Tianyue Electronics Co. Ltd., Guangzhou, China). After 6 mins, 150 μL of 10% AlCl_3_·6H_2_O solution was added, and the mixture was left to react for 5 mins before adding 0.5 mL of 1 mol/L NaOH solution and 275 μL water. The absorbance was measured at a wavelength of 510 nm. The total flavonoid content was determined from a standard curve using catechin as the standard. The concentration of catechin standard solution was 0.01–0.5 mg/mL. The standard curve equation was: y = 0.10x + 0.0464; *R*^2^ = *0.9895*. x(mg/mL) was the concentration of catechin; y was the absorbance of catechin.

#### 2.3.2 Determination of total phenols contents

Total polyphenols contents in fruit vinegars were measured by the Folin–Ciocalteau method (Spagnuolo et al., 2021, Singleton and Rossi, 1965). An aliquot of 0.5 mL of fruit vinegar was mixed with 0.5 mL of Folin reagent (diluted 5 times), followed by incubation for 3 mins. Then, 1 mL of 15% Na_2_CO_3_ was added. The absorbance at 765 nm was measured after incubation in the dark for 30 mins, using a microplate reader (Thermo, America). Measures were performed in triplicate. The total polyphenols content was determined from a standard curve using gallic acid (0.01–0.6 mg/mL) as a standard and expressed. The standard curve equation was: y = 4.97x + 0.338; *R^2^ = 0.9947.* × (mg/mL) was the concentration of gallic acid; y was the absorbance of gallic acid.

### 2.4 Determination of antioxidant activity

#### 2.4.1 Determination of DPPH free radical scavenge capacity

The capacity to scavenge the DPPH free radical was determined according to the method by Lertittikul (Lertittikul, Benjakul, & Tanaka, 2007). Fruit vinegar was mixed with 0.1 mmol/L of DPPH ethanol solution. The OD value at 517 nm was measured after incubation in the dark for 30 mins, using an ultraviolet–visible spectrophotometer (Ruili Analytical Instrument Co. Ltd., Beijing, China). The scavenging effect was calculated as follow (Eq. (1)):

(1) $$\text{DPPH free radical scavenging ability}\left(\%\right)=\left[1-\left({\text{A}}_{\text{sample}}-{\text{A}}_{\text{control}}\right)/{\text{A}}_{\text{blank}}\right]\times 100\%$$

A_sample_ was the OD value of the ethanol solution with fruit vinegar and DPPH, A_control_ was the OD value of the ethanol solution without fruit vinegar and DPPH. A_blank_ was the OD value of the fruit vinegar replaced with distilled water. Distilled water and ethanol solution were as reference solution to adjust to zero.

#### 2.4.2 Determination of ferric ion reducing antioxidant power (FRAP)

The FRAP of fruit vinegars were analyzed to the method described previously (Benzie, & Strain, 1996). The FRAP working solution was produced by mixing 20 mmol/L ferric chloride hexahydrate with 10 mmol/L TPTZ and 300 mmol/L pH3.6 sodium acetate buffer. Then, fruit vinegar, preheat at 37 ℃ for 2 mins, was mixed with FRAP working solution and 300 μL distilled water. The absorbance at 593 nm was monitored after 4 mins using a spectrophotometer. The antioxidant activity of each sample was calculated using the following formula (Eq. (2)):

(2) $$\text{Iron ion reduction capacity}\left(\%\right)\;=\;\left({\text{A}}_{\text{sample}}-{\text{A}}_{\text{control}}\right)\;/{\;\text{A}}_{\text{blank}}\times 100\%$$

A_sample_ was the OD value of the fruit vinegar sample, A_control_ was the OD value of the same volume of distilled water instead of ferric chloride, and A_blank_ was the OD value of the distilled water.

#### 2.4.3 2,2′-Azinobis-(3-ethylbenzthiazoline-6-sulphonate) (ABTS) assay

The trolox equivalent antioxidant capacity (TEAC) assay is based on the ability of the antioxidant present in A_sample_ to scavenge the radical cation ABTS by spectrophotometric analysis. ABTS^+^ radicals were prepared by reacting 7.4 mM ABTS solution with 2.6 mM K_2_S_2_O_8_ solution, stored in the dark at normal temperature for 12 h. Then, the ABTS^+^ solution was diluted by phosphate buffer solutions (10 mmol/L, pH 7.4) to an absorbance value near 0.7 ± 0.02 at 734 nm.(Albano, 2002) The samples were mixed with the ABTS^+^ solution and kept at room temperature for 6 mins. The antioxidant activity of fruit vinegar was calculated using the following formula (Eq. (3)):

(3) $${\text{ABTS}}^{+}\;\text{free radical scavenging rate}\;\left(\%\right)\;=\left({\text{A}}_{\text{control}}-{\text{A}}_{\text{sample}}\right)/{\text{A}}_{\text{control}}\times 100\%$$

A_sample_ was the OD value of the fruit vinegar sample, and A_control_ was the OD value of fruit vinegar replaced with alcohol.

### 2.5 Odor analysis

#### 2.5.1 Electronic nose analysis

2.5 mL of fruit vinegar and water were placed into a 20 mL headspace bottle. The sample was equilibrated for 56 mins in a water bath at 50 ℃ to development of headspace before analysis. Then, sample needle was used to perforate the seal of the vial and to absorb the air accumulated inside it at a flow rate of 300 mL/min during the measurement (Xu, Yu, Liu, & Zhang, 2016). Data were recorded every second by the computer, and the experiment lasted 60 s. Each sample was analyzed in triplicate and the average of the results was used for subsequent statistical analysis.

#### 2.5.2 Gas chromatography–Mass spectrometry (GC–MS) analysis

The volatile aroma compounds of fruit vinegars were analyzed according to Özen et al. (2019) with some modifications. Analyses were conducted using an Agilent GC system coupled to an Agilent inert quadrupole mass spectrometer. A mixture of 5 mL fruit vinegar, equal amount of distilled water and 1 g NaCl was performed at 50 °C for 30 mins and extracted by extraction column for 0.5 h. The splitless inlet temperature was 250 °C. Separation was performed on an Agilent HP-5MS capillary column (30 m × 250 μm, 0.25 μm film thickness). The carrier gas was high-purity helium (Purity ≥99.999%) at a constant flow rate of 1 mL/min. Using programmed temperature increase, the column oven temperature was initially set at 35 °C for 3 mins, and then increased to 150 ℃ at a rate of 9 ℃/min and then to 250 °C at a rate of 6 ℃/min and held for 2 mins. Mass spectrometry conditions: the ion source was an EI source and maintained at 230 °C, the MS quadrupole temperatures was maintained at 150 °C and the collision energy was 70 eV. Collect data in full scan mode, the scan range was 50 ∼ 500 Da and the scan speed was 3125 μ/s.

### 2.6 Polyphenolic compounds analysis of fruit vinegar

Pass 5 mL of fruit vinegar through a pre-activated C18 solid phase extraction column (pre-activation conditions: 3 mL methanol, 3 mL distilled water), then rinse with 3 mL distilled water, 3 mL methanol, 5 mL methanol containing 5% ammonia water. The target compound was eluted, and finally subjected to rotary evaporation, re-dissolved in 1 mL of methanol, filtered with a 0.22 μm filter membrane, and determined by A Symmetry C18 column (4.6 × 250 mm, 5 μm, waters, Ireland) on a SHIMADZU liquid chromatography with a diode array detector (Shimadzu Co., Kyoto, Japan). Chromatographic conditions: mobile phase A was 0.1% formic acid aqueous solution, mobile phase B was methanol, injection volume was 3 μL, detection wavelength was 280 nm, flow rate was 0.2 mL/min, column temperature was 25 °C. Gradient elution conditions: 0 ∼ 8 min was 95% ∼ 65% A, 8 ∼ 9 min was 65% ∼ 50% A, 9 ∼ 10 min was 50%∼20% A, 10 ∼ 12 min was 20% ∼ 5% A, 12 ∼ 13 min was 5% ∼ 95% A, continue to run for 4 mins to equilibrate the column, and the total running time was 18 mins (Wu et al., 2022, Li et al., 2022).

Mass spectrometry conditions: negative ion electrospray ionization (ESI-); ion source temperature was 300 ℃; capillary voltage was 4 kV; the temperature of the quadrupole rod of the primary mass spectrometer and the secondary mass spectrometer were both 100 ℃; the sheath gas temperature was 350 ℃, sheath flow velocity was 11 L/min, drying gas velocity was 10 L/min, atomizer pressure was 40 psig, ion scanning range was 50 ∼ 500 *m*/*z*. MassHunter Data and MassHunter Qualitative were used for qualitative and quantitative analysis of data.

### 2.7 The inhibitory effect of fruit vinegar and its main components on AGEs formation

5 mg/mL BSA and 36 mg/mL glucose in a phosphate buffer solution were mixed with 0.2, 1 mL of fruit vinegar or 0.2, 1 mg/mL of aminoguanidine (AG) at pH 7.4 and heated for 5 days in a 50 ℃ thermostat. The amount of free fluorescent AGEs was determined on 2 d and 5 d using an F-4500 luminescence spectrometer (Shimadzu, Japan) at excitation/emission wavelengths of 370/440 nm (Wu et al., 2021a). The inhibition rate on AGEs formation was calculated according to the following formula (Eq. (4)):

(4) $$\text{Inhibition rate}\left(\%\right)=\;\left({\text{F}}_{\text{control}}-{\text{F}}_{\text{sample}}\right)/\left({\text{F}}_{\text{control}}-{\text{F}}_{\text{blank}}\right)\times 100\%$$

F_sample_ was the fluorescence value of the reaction solution with fruit vinegar; F_blank_ was the fluorescence value of the unheated sample, and F _control_ was the fluorescence value of the reaction solution without fruit vinegar.

### 2.8 MTT (3-[4,5-dimethylthiazole-2-yl]-2,5-diphenyltetrazolium bromide) assay

MTT assay was used for the analysis of cell cytotoxicity inhibition. Caco-2 cell was taken in each well of 96 well plates at a density of 8 × 10^3^ cells/well and incubated overnight in a humidified carbon dioxide incubator at 37 ℃ and 5% CO_2._ A varying concentration of N-ε-carboxymethyllysine (CML), orange vinegar and its main components catechin, epicatechin, and *p*-coumaric acid were added to each well. Following incubation of the plates at 37 ℃ and 5% CO_2_ for 12 h, the medium was discarded and 150 μL of MTT solution (0.5 mg/mL) was added to each well. After 2 h incubation, MTT solution was removed and dimethyl sulfoxide (DMSO) (150 μL/well) was added. Then, absorbance values were measured at 490 nm using microplate reader (Wu et al., 2021b). The cell survival rate was calculated using the following formula (Eq. (5)).

(5) $$\text{Cell survival rate}\left(\%\right)=\left[{\text{A}}_{\text{sample}}-{\text{A}}_{\text{blank}}\right]\;/\;\left[{\text{A}}_{\text{control}}-{\text{A}}_{\text{blank}}\right]\times 100\%$$

A_sample_ was the OD value of the sample group, A_control_ was the OD value of the sample replaced with MEM and A_blank_ was the OD value of the cell-free and sample-free group.

### 2.9 Determination of ROS in Caco-2 cells

The Caco-2 cells were prepared for the assay by incubating with 3 mmol/L CML, 3 mmol/L CML + 20 times diluted orange vinegar, 3 mmol/L CML + 0.1 μmol/L catechin, 3 mmol/L CML + 0.1 μmol/L epicatechin and 3 mmol/L CML + 0.1 μmol/L coumaric acid for 24 h, respectively. Cell suspension were taken on coverslip which placed at the bottom of the 6-well plates and incubated in a humidified carbon dioxide incubator at 37 ℃ and 5% CO_2_ for 8 h to fix the cells. After washing three times with PBS, they were incubated with 1 mL of 5 mM DHE (dissolved in DMSO) at 37 ℃ for 30 mins in the dark (Gatliff et al., 2014, Liao et al., 2023). Then, the cells were washed using PBS buffer and stained by 4′,6-diamidino-2-phenylindole (DAPI) for 10 mins in the dark. Anti-fluorescence quenching agent was added and photographed using a fluorescence microscope.

### 2.10 Determination of mitochondrial membrane potential in Caco-2 cells

Cell suspension after incubation with samples for 24 h were taken on coverslip at the bottom of the 6-well plates and incubated at 37 ℃ and 5% CO_2_ for 8 h to fix the cells. Working assay solution was prepared by adding 50 μL of 5,5,6,61-tetrachloro-1,1′,3,3′-tetraethyl-imidacarbocyanine iodide (JC-1) (200X) for every 8 mL of ultrapure water and added 2 mL of JC-1 staining buffer (5X) to become the JC-1 staining working solution. After incubation with 1 mL JC-1 staining solution at 37 °C in the cell incubator for 20 mins, caco-2 cells were washed with JC-1 staining buffer 2 times and then incubated with DAPI staining solution for 10 mins in the dark. The cover slides were mounted with 2 mL cell culture medium and then observed under a fluorescence microscope.

### 2.11 Western blot analysis

RAGE, NADPH, p38 mitogen-activated protein kinase (MAPK), p-p38MAPK, forkhead box protein O1 (FOXO1), B-cell lymphoma (Bcl)-2, BCL-2-associated X (Bax), Caspase-3 and Caspase-9 proteins were measured by western blot analysis according to Wu et al (Wu et al., 2021). The Caco-2 cells were prepared for the assay by incubating with 3 mmol/L CML, 3 mmol/L CML + 20 times diluted orange vinegar, 3 mmol/L CML + 0.1 μmol/L catechin, 3 mmol/L CML + 0.1 μmol/L epicatechin and 3 mmol/L CML + 0.1 μmol/L coumaric acid for 24 h, respectively. The cells treated with the fruit vinegar were rinsed with sterile PBS and lysed using cell protein extraction reagent for 4 mins. After repeatedly pipetted during an ice bath for 30 mins, samples were centrifuged at 10,000 rpm for 7 mins at 4 °C to collect the supernatant. 40 μg proteins were electrophoresed on 10 % polyacrylamide gel and transferred to polyvinylidene fluoride (PVDF) membranes. The converted membrane was blocked with 5 % skim milk (1 M Tris-HCl, pH 7.5, 150 mM NaCl and 0.1 % Tween-20) for 1 h at room temperature and incubated overnight at 4 ℃ with primary antibodies in TBST (GAPDH, 1:10,000; RAGE, 1:1000; p65NF-κB, 1:2000; p38MAPK, 1:2000; p-p38MAPK, 1:1000), followed by incubation with diluted secondary antibody (1:10,000) for 30 mins. The enhanced chemiluminescence (ECL) mixed solution (ASPEN, South Africa) was added dropwise to collect images and the optical density value was analyzed using the AlphaEase FC software.

### 2.12 ELISA to detect interleukin-6 (IL-6) and tumor necrosis factor-α (TNF-α) levels in the cell culture supernatant

The Caco-2 cells were prepared for the assay by incubating with fruit vinegar at a density of 3 × 10^5^ cells per well for 24 h. The secretion levels of inflammatory cytokines (TNF-α and IL-6) in the cell culture supernatant was determined by ELISA kits (KeyGEN, Nanjing, China). The OD value of the ELISA plate was measured at 405 nm by a microplate reader according to the manufacturer's instructions.

### 2.13 Statistical analysis

Origin 8.0 software was used to perform the data of samples. The results were expressed as the means ± standard deviation (SD). The significant level was expressed as *P < 0.05* if there were differences between groups.

## 3 Results and discussion

### 3.1 Total flavonoid and polyphenols contents in fruit vinegars

Our research showed that the total flavonoid contents of eight fruit vinegars had a certain difference (Fig. S1). Among them, kiwi vinegar, orange vinegar and persimmon vinegar had higher flavonoid content, and the content reached 0.74 ± 0.08 mg/mL, 0.62 ± 0.03 mg/ mL and 0.34 ± 0.03 mg/mL, respectively. Mango vinegar had the lowest total flavonoid content, only 0.05 ± 0.01 mg/mL. Regarding the content of polyphenols, it was basically the same as the content of total flavonoids. The content of polyphenols in orange vinegar, kiwi vinegar, and persimmon vinegar were also higher, reaching 0.34 ± 0.0096 mg/mL, 0.33 ± 0.0097 mg/mL and 0.22 ± 0.0057 mg/mL, respectively. The content of polyphenols in other fruit vinegars was around 0.1 mg/mL, except for apple cider vinegar, which was only 0.012 ± 0.0007 mg/mL.

### 3.2 Antioxidant activity of different fruit vinegars

In this study, three methods were used to evaluate the antioxidant activities of eight kinds of brewed fruit vinegars. As shown in Fig. S2, the scavenging rate of DPPH free radicals was basically between 25% and 30%. Among them, DPPH free radical scavenging rate of persimmon vinegar was 32.13 ± 1.38%, which was higher than others. The order of different fruit vinegars on the scavenging rate of FRAP free radicals was: orange vinegar > persimmon vinegar > pear vinegar > grape vinegar > apple cider vinegar > mango vinegar > watermelon vinegar. The maximum inhibition rate was orange vinegar, reaching 49.86 ± 1.87%. There were significant differences in the results of ABTS free radical scavenging rate. The order of inhibition rate was: mango vinegar > pear vinegar > persimmon vinegar > grape vinegar > orange vinegar > kiwi vinegar > watermelon vinegar > apple cider vinegar. Mango vinegar had the highest scavenging rate of ABTS free radicals up to 83.28 ± 0.76%. It was probably because the polyphenols in fruit vinegar had hydroxyl groups, which can combine with unstable molecules to generate stable substances and finally achieved anti-oxidation effect (Brglez Mojzer, Knez Hrnčič, Škerget, Knez, & Bren, 2016, Feng et al., 2023). In terms of comprehensive antioxidant indicators, the scavenging ability of different free radicals was different, which may be caused by the different types and contents of polyphenols in different fruit vinegars (Olszowy, 2019). But from the overall results, orange vinegar and persimmon vinegar were in the leading position in each index.

### 3.3 Odor analysis

#### 3.3.1 Electronic nose analysis

**Fig. 1:**
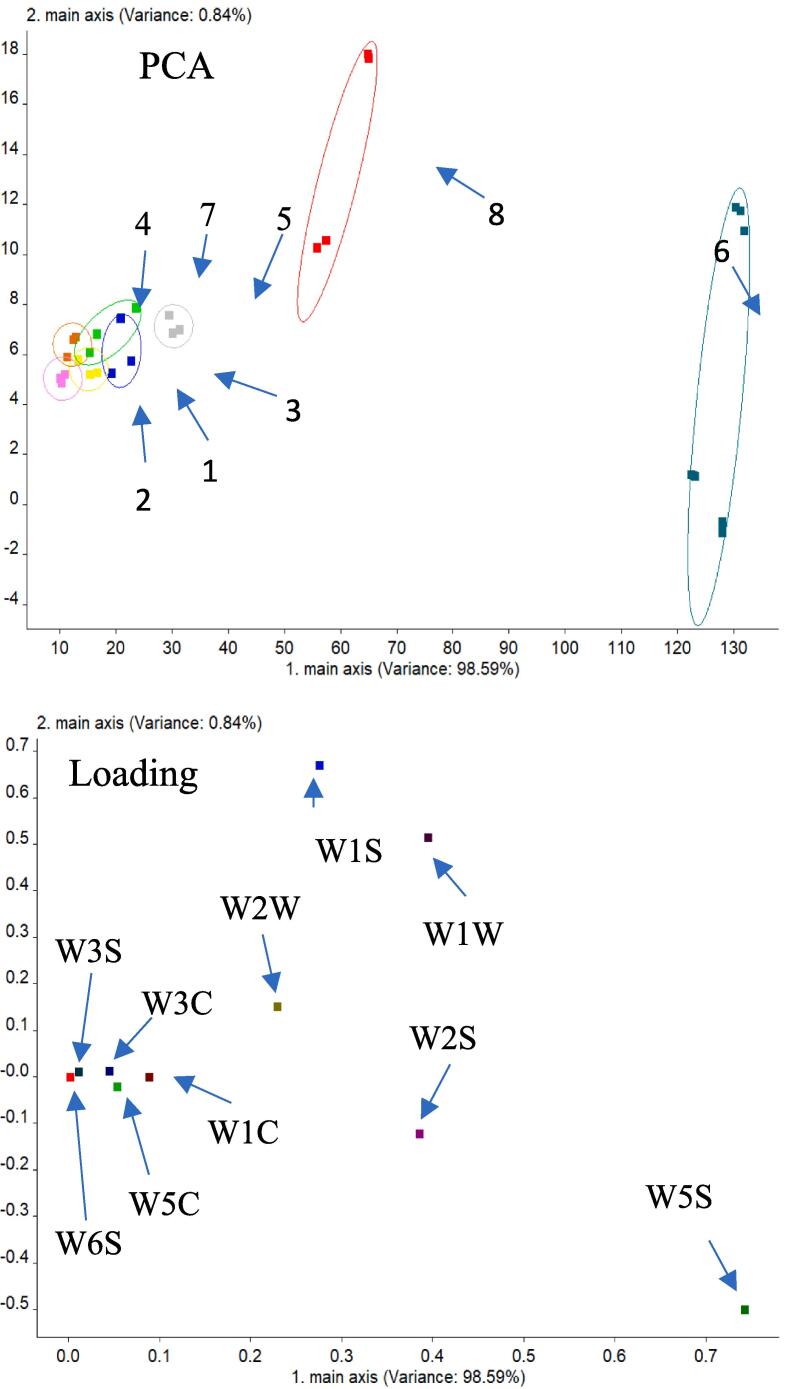
Analysis of fruit vinegar electronic nose. W1C: Aromatic ingredients, benzene; W5S: High sensitivity, very sensitive to nitrogen oxides; W3C: Sensitive fragrance, ammonia; W6S: Mainly selective to hydrides; W5C: Aromatic components of short-chain alkanes; W1S: Sensitive to methyls; W1W: Sensitive to sulfides; W2S: Sensitive to alcohols, aldehydes and ketones; W2W: Aromatic ingredients; W3S: Sensitive to long chain alkanes.

The E-nose, an analytical instrument that mimics the human olfactory system, is a nondestructive method whose detection process is simple and fast (Zhu et al., 2020). Fig. 1A showed the principal component analysis (PCA) of aroma components of eight fruit vinegars using the E-nose. The total variance in the contribution of the first two principal components (PCs) was 99.43% (PC1 and PC2 were 98.59% and 0.84%, respectively), which indicates that PC1 and PC2 reflected much information of the overall characteristics of the samples (Xu, Yu, Liu, & Zhang, 2016). According to the discreteness of the response values, PCA could be used to distinguish aroma among the eight vinegars. The greater the degree of dispersion between data acquisition points, the better differentiation of the groups. There was no obvious overlap of volatile flavour data collection points among mango, grape and kiwi vinegars, which indicated that they could be better distinguished. It could be seen that the data points of persimmon vinegar, apple vinegar, orange vinegar and pear vinegar had a certain overlap, which was no significantly different in the flavor components.

The contribution rate of 10 sensors of E-nose to PCA principal component analysis of samples was shown in Fig. 1B. Loading analysis results showed that sensors W5S, W1W, and W2S contribute significantly to PC1, indicating that the first principal component was mainly nitrogen oxides, sulfides, alcohols, aldehydes and ketones. W1S, W1W, and W2W had a large contribution rate to PC2, indicating that the second principal component was mainly methyl compounds, sulfides, and organic sulfides. Based on the above analysis, it was found that the main flavor substances of fruit vinegar were aldols, which just provided a good smell for fruit vinegar (Parker, 2015).

#### 3.3.2 GC–MS analysis

In addition to the electronic nose analysis, a more detailed study on the flavor composition of the fruit vinegars was performed through gas chromatography–mass spectrometry method. As shown in Table 1, pear vinegar, watermelon vinegar, kiwi vinegar, orange vinegar, persimmon vinegar, grape vinegar, apple vinegar and mango vinegar contained 11, 16, 16, 15, 6, 12, 8, 14 flavor substances respectively, including acetic acid, isovaleric acid, 2,4-Di-*tert*-butylphenol, pelargonic acid, dihydro-5-pentyl-2(3H)-furanone, *trans*-2-decenal, 9-hexadecenoic acid ethyl ester, ethyl hexadecanoate, ethyl palmitate, benzaldehyde, 2-phenylethyl formate and capric acid.

**Table 1:** Analysis of flavor components of fruit vinegar.

	Acid	Apple cider vinegar(%)	Watermelon vinegar (%)	Orange vinegar (%)	Persimmon Vinegar (%)	Kiwi Vinegar (%)	Grape vinegar (%)	Pear vinegar (%)	Mango vinegar (%)
1	Acetic acid	24.79	0.7	6.73	2.37	10.31	4.88	7.66	3.30
2	Capric acid	–	–	–	–	–	–	–	5.11
3	Palmitic acid	0.35	0.07	0.44	–	0.18	12.95	0.31	0.3
4	Undecanoic acid	0.14	0.93	–	–	0.06	0.2	0.34	0.04
5	Pentadecanoic acid	–	0.46	–	–	0.05	0.16	0.13	0.14
6	Pelargonic acid	–	12.42	0.48	–	–	–	–	–
7	Lauric acid	–	–	2.2	–	–	0.63	–	0.31
8	Octanoic acid	–	–	1.32	–	–	–	–	–
9	Oleic acid	–	–	1.36	–	0.49	1.12	–	0.74
10	Isovaleric acid	–	–	–	22.29	–	–	6.81	–
	Total	25.28	14.58	12.53	24.66	11.09	19.94	15.25	9.94

	**Ester**								
11	9-Hexadecenoic acid ethyl ester	–	3.83	8.43	1.13	1.24	9.06	2.14	4.04
12	Ethyl hexadecanoate	5.12	–	7.4	–	–	–	2.45	4.2
13	Ethyl oleate	1.38	0.7	1.62	–	–	–	0.89	–
14	formic acid -2-phenyl ethyl ester	1.16	2.9	–	–	–	–	3.00	27.3
15	Ethyl palmitate	–	3.63	–	2.92	3.08	6.21	–	–
16	Elaidic acid ethyl ester	–	0.67	2.28	–	0.66	2.47	–	1.4
17	Ethyl Decanoate	–	–	1.94	–	1.5	–	–	–
18	Isoamyl acetate	–	–	6.33	–	–	–	–	–
	Total	7.66	11.73	28	4.05	6.48	17.74	8.48	36.94

	**Alcohol**								
19	Phenylethanol	62.25	55.65	51.25	66.57	23.7	43.11	64.27	42.6
	Total	62.25	55.65	51.25	66.57	23.7	43.11	64.27	42.6

	**Phenol**								
20	2,4-Di-*tert*-butylphenol	3.59	3.03	2.02	2.08	0.34	9.84	7.93	9.35
	Total	3.59	3.03	2.02	2.08	0.34	9.84	7.93	9.35

	**Aldehyde**								
21	Trans-2-decenal	0.35	–	–	–	48.82	–	–	–
22	Hexadecaldehyde	–	0.84	–	–	–	–	–	–
23	*N*-octanal	–	–	–	–	4.26	–	–	–
24	Nonanal	–	–	–	–	4.24	–	–	–
25	Trans-2-dodecenal	–	–	–	–	0.96	–	–	–
26	Benzaldehyde	–	–	–	–	–	10.19	–	1.2
	Total	0.35	0.84	0	0	58.28	10.19	0	1.2

	**Ketone**								
27	Geranyl acetone	–	3.05	–	–	–	–	–	–
28	β-ionone	–	1.33	2.13	–	–	–	–	–
29	Phyton	–	–	–	–	0.13	–	–	–
30	Dihydro-5-pentyl-2(3H)-furanone	–	9.79	–	–	–	–	–	–
	Total	0	14.17	2.13	0	0.13	0	0	0

A low concentration of acid will give fruit vinegar a light and pleasant fragrance, but when the concentration is too high, it will have a negative impact on the aroma quality of fruit vinegar (Duan, Liu, Lv, Wu, & Wang, 2020). As shown in Fig. S3, the main components in fruit vinegar were alcohols, acids, and esters, among which alcohols were only benzene ethanol. Phenethyl alcohol has soft and lasting rose and honey aroma, which has positive effect on the formation of fruit wine aroma (Margetts, 2004). Most fruit vinegars contained a relative alcohol content of 50–60%. Among them, the content of alcohol in persimmon vinegar was the highest, reaching 66.57%. The relative content of acid in fruit vinegar was about 10%-25%, including acetic acid, palmitic acid, undecanoic acid, pentadecanoic acid and oleic acid. The ester compounds are the important substances constituting the aroma of fruit wine and give fruit wine a unique fruit aroma (Ickes, 2017). The main ester compounds in fruit vinegar were 9-hexadecenoic acid ethyl ester, ethyl hexadecanoate, ethyl oleate, formic acid −2- phenyl ethyl ester and ethyl palmitate.

### 3.4 Composition analysis of fruit vinegar

The compositions of fruit vinegars were effectively analyzed by liquid chromatography-mass spectrometer. It was found that the eight fruit vinegars basically contained 7 kinds of polyphenols: ferulic acid, *p*-coumaric acid, vanillic acid, caffeic acid, chlorogenic acid, catechin and epicatechin. As shown in Table 2, the relative content of polyphenol in each vinegar was different, but they all contained high contents of catechin. Pear vinegar, mango vinegar, kiwi vinegar, grape vinegar, persimmon vinegar and watermelon vinegar contained vanillic acid with relatively high content. Apple and orange vinegar had a high content of ferulic acid. In general, orange vinegar had the highest amount of polyphenol compared to other vinegars.

**Table 2:** Composition analysis of phenolic compounds in fruit vinegar.

Peak	tR (min)	Main compound	[M–H]– (*m*/*z*)	Typical MS^2^ ions (*m*/*z*)	Apple ×10^−7^ (*mg/mL*)	Watermelon ×10^−7^ (*mg/mL*)	Mandarin Orange ×10^−7^ (*mg/mL*)	Persimmon ×10^−7^ (*mg/mL*)	Kiwi fruit ×10^−7^ (*mg/mL*)	Grape ×10^−7^ (*mg/mL*)	Pear ×10^−7^ (*mg/mL*)	Mango ×10^−7^ (*mg/mL*)
1	5.891	Catechin	289.07	245.08	24.6	159	486	2.5	127	276	72.7	185
2	6.488	Chlorogenic acid	353.08	191.05	1.35	1.21	1.44	0	4.91	4.19	13.5	2.81
3	6.773	Vanillic acid	167.03	108.02, 123.04	2.95	23.7	1.32	0.94	32	23.3	88.2	23.8
4	7.085	Caffeic acid	179.03	135.04	1.79	1.25	11.4	0.381	42.7	21.2	2.18	3.71
5	7.682	Epicatechin	289.07	245.08	1.21	0	355	0.55	39.4	9.49	4.34	0
6	8.914	*p*-Coumaric acid	163.02	119.04	1.41	11.8	12.9	0.279	1.67	1.54	3.8	19.8
7	9.515	Ferulic acid	193.05	134.03, 149.05	43.2	7.2	4.64	1.92	1.98	8.31	3.43	15

### 3.5 The inhibitory effect of fruit vinegar and its main components on AGEs formation

As shown in Fig. S5, orange vinegar, kiwi vinegar, grape vinegar, persimmon vinegar and mango vinegar had better inhibitory effects on the formation of AGEs than the positive control AG. Their inhibition rates were 59.69 ± 4.24/62.65 ± 6.03%, 51.64 ± 5.03/55.23 ± 3.02%, 47.00 ± 1.04/51.99 ± 0.80%, 48.50 ± 3.38/57.05 ± 1.36%, 49.02 ± 1.54/51.20 ± 1.52% (0.2/1 mL), respectively. Among them, orange vinegar had the best inhibitory effect, potentially because it had relatively high content of total polyphenols and flavonoids according to the previous results, which can effectively capture the dicarbonyl compounds to inhibit the formation of AGEs (Lo, Hsiao, & Chen, 2011). Meanwhile, compared with other fruit vinegars, orange vinegar had better effect on antioxidant activity, which correlated positively with the antiglycation activity (Harris et al, 2011).

### 3.6 MTT

The ability of orange vinegar and its main components to protect Caco-2 cells from AGEs-induced damage was determined using the MTT assay. When cells were exposed to 3 mM CML or higher, a significant (*p* < 0.05) concentration-dependent decrease in cell viability was observed in Fig. S6. MTT assay showed toxic mitigation effects of orange vinegar and its main components, where catechin was the best inhibitor, followed by *p*-coumaric acid and epicatechin, while orange vinegar had limited cytotoxicity inhibition effects. After catechin was incorporated with CML, cell survival rate was increased from 48.15 ± 1.34% to 73.19 ± 1.32%. According to the MTT experiment, CML 4 mmol/L, orange vinegar diluted 20 times, catechin, epicatechin, and *p*-coumaric acid 0.1 μmol/L were chosen as tested concentration.

### 3.7 Effects of orange vinegar and its main components on ROS level and mitochondrial membrane potential in Caco-2 cells

**Fig. 2:**
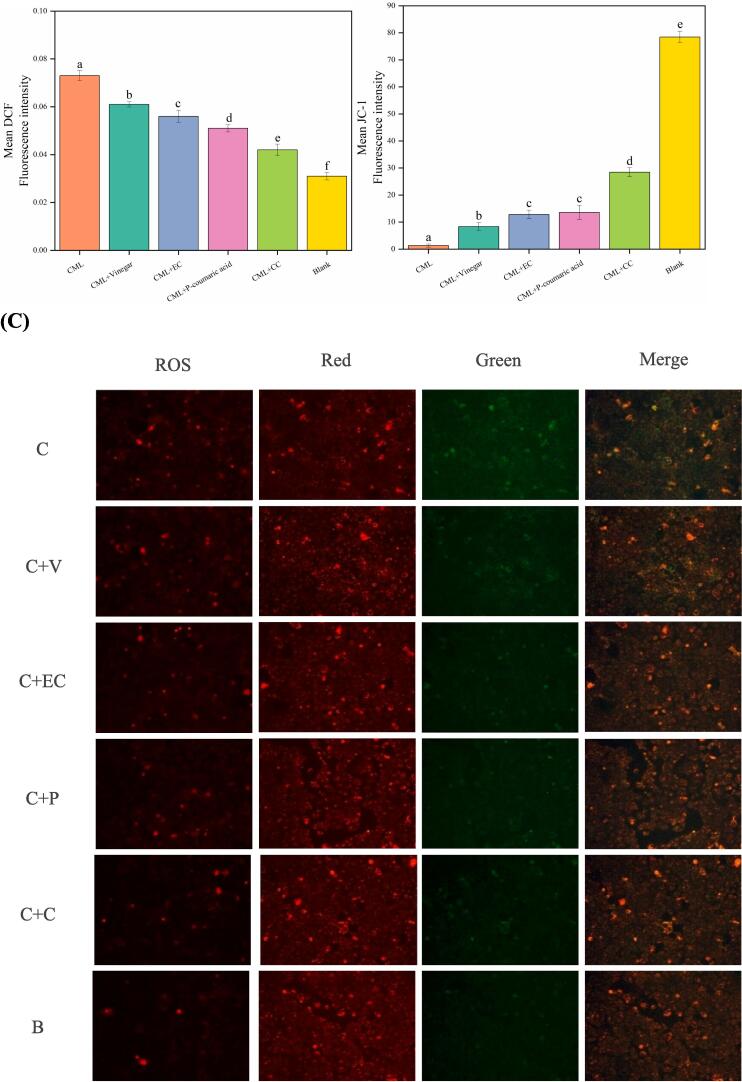
Effects of orange vinegar and its main components on ROS and mitochondrial membrane potential in Caco-2 cells. A, The fluorescence intensity of DCF; B, The fluorescence intensity of JC-1; C, Detection of DCF and JC-1 signals in Caco-2 cells by fluorescence microscope. Green: JC-1 monomers; Red: JC-1 aggregates. C: CML; C + V: CML + vinegar; C + EC: CML + epicatechin; C + P: CML + *p*-coumaric acid; C + CC: CML + catechin; B: blank. Different letters indicated significant differences (*P < 0.05*). (For interpretation of the references to color in this figure legend, the reader is referred to the web version of this article.)

Fluorescence spectrophotometer was used to determine the level of ROS in the Caco-2 cells. As shown in Fig. 2, CML significantly increased the level of ROS in Caco-2 cells, indicating that CML could induce intracellular ROS generation. An increase of ROS cause oxidative degeneration of macromolecules by transferring electrons and cause damage to polyunsaturated fatty acids, proteins and DNA, which successively mediate pathological processes such as atherosclerosis, diabetes, neurodegeneration and inflammation (Zhang et al., 2016). The addition of orange vinegar, catechin, epicatechin and *p*-coumaric acid significantly inhibited the expression of ROS (*p* < 0.05) and the sequence of ROS fluorescence intensity was: CML > CML + Vinegar > CML + epicatechin > CML + *p*-coumaric acid > CML + catechin > Blank.

The mitochondrial membrane potential was measured by JC-1 staining and detected using a fluorescence microscope. Cells exposed to CML showed increased conversion of JC-1 aggregates (red) to JC-1 monomers (green), which proved that the cell membrane potential had dropped and showed that CML induced mitochondrial dysfunction in Caco2-cells (Liu et al., 2020). Following treatment with orange vinegar, catechin, epicatechin and *p*-coumaric acid, the cells showed slightly green fluorescence. Compared with the CML group, orange vinegar and its main components exposure effectively improved the decrease in membrane potential caused by CML (*p <* 0.05), further reducing early cell apoptosis. In a general way, the results obtained for mitochondrial membrane potential and ROS level (Fig. 2) were in line with the one achieved for MTT assay (Fig. S6), with the catechin demonstrating the best results.

### 3.8 Effects of orange vinegar and its main components on RAGE, NADPH, p38MAPK, p-p38MAPK, FOXO1, Bcl-2, Bax, Caspase-3, Caspase-9 expression

**Fig. 3:**
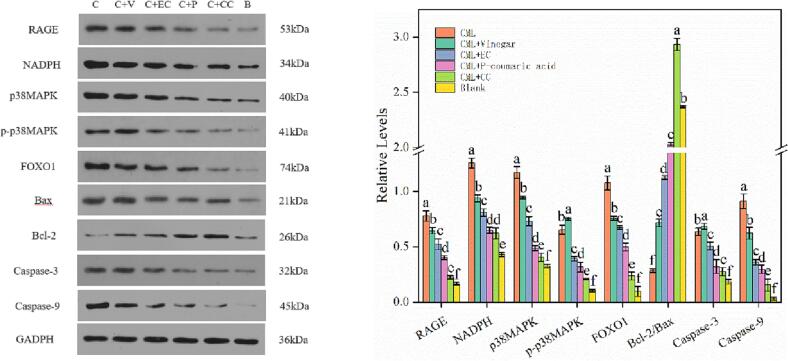
Effects of orange vinegar and its main components on expression of RAGE, NADPH, p38MAPK, p-p38MAPK, FOXO1, Bcl-2, Bax, Caspase-3, Caspase-9. Different letters indicated significant differences (*P < 0.05*). (For interpretation of the references to color in this figure legend, the reader is referred to the web version of this article.)

We investigated whether AGEs and polyphenols treatment would alter expression of apoptosis-related proteins in the Caco-2 cells by Western Blot. As shown in Fig. 3, the expression levels of RAGE, NADPH, p38MAPK and p-p38MAPK significantly increased in Caco-2 cells treated with CML, While the levels decreased by mixing with orange vinegar, catechin, epicatechin and *p*-coumaric acid. AGEs combined with RAGE to activate NADPH oxidase, which resulted in the increase of intracellular ROS, then activated downstream MAPK pathway, which is essential for the expression of activation-induced inflammatory mediators (Tobon-Velasco, Cuevas, & A Torres-Ramos, 2014). Therefore, catechin, epicatechin and *p*-coumaric acid could protect Caco-2 cell from RAGE-MAPK-NF-κB signaling by suppressing AGEs-induced oxidative stress response. After cells treated with CML, the levels of FOXO1, caspase-3, -9, and Bax increased and the level of Bcl-2 decreased, demonstrating that CML activated cell apoptosis (Qiao et al., 2012, Zhang et al., 2022). Meanwhile, with catechin, epicatechin and *p*-coumaric acid treatments, the levels of caspase-3, -9 and FOXO1 decreased and the relative level of Bcl-2/Bax increased, which means that catechin, epicatechin and *p*-coumaric acid could protect Caco-2 cell from apoptosis. Xie et al. found that ferulic acid (FA) protected ARPE-19 cells from damage induced by hydrogen peroxide (H_2_O_2_). Adding FA (5 mmol/L) could reduce the expression of Bax and cleaved caspase-3 proteins and increased the expression of Bcl-2 protein in ARPE-19 cell, which was the same as our research conclusion (Xie, Jin, Zhu, Zhou, Du, & Jin, 2020).

### 3.9 Effects of orange vinegar and its main components on the levels of IL-6 and TNF-α in cell supernatant

In order to explore the protective effect of orange vinegar and its main components on Caco-2 cells, the secretion of inflammatory factors was detected. As shown in Fig. S7, we found that catechin, epicatechin and *p*-coumaric acid effectively protected Caco-2 cells and significantly reduced the expression of inflammatory factors induced by CML, while orange vinegar had little effect. The order of protection for Caco-2 cells was: catechin > coumaric acid > epicatechin. CML significantly induced the secretion of inflammatory factors and increased the expression of TNF-α and IL-6 from the initial 1.32 ± 0.84 pg/mL and 2.28 ± 1.31 pg/mL to 56.87 ± 1.61 pg/mL and 17.87 ± 2.32 pg/mL, increasing nearly 43 to 8 times. The expression of TNF-α in the group supplemented with orange vinegar, epicatechin, *p*-coumaric acid, and catechin were 45.92 ± 1.68 pg/mL, 32.05 ± 4.37 pg/mL, 21.34 ± 3.97 pg/mL, and 17.38 ± 4.82 pg/mL, respectively. Research by Jo N Y found that Paulownia fluff extract (PTE) had antioxidant and anti-inflammatory effects and significantly inhibited the expression of inflammatory factors (TNF-α, IL-6) (Jo & Kim, 2019). Yang et al. found that apple polyphenols improved the antioxidant capacity of grass carp, protected grass carp from inflammation, and reduced the expression of IL-6 (Yang et al., 2021). Chen et al. found that lotus leaf flavonoids extract significantly reduced the levels of IL-6, IL-12 and TNF-α with the increase in concentration (Chen et al., 2020). Our results indicated that orange vinegar and its main components could reduce the expression of inflammatory factors induced by CML.

## 4 Conclusion

**Fig. 4:**
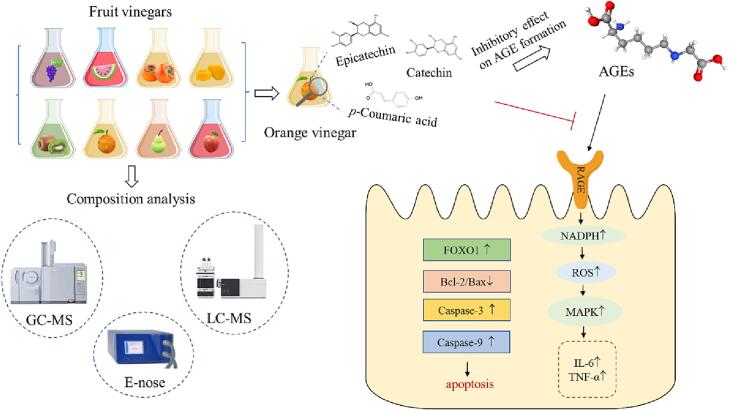
Possible protective mechanism of orange vinegar and its main components on cytotoxicity in Caco-2 cell. (For interpretation of the references to color in this figure legend, the reader is referred to the web version of this article.)

In summary, GC–MS, LC-MS and E-nose methods were applied in order to assess the vinegar’s (watermelons, grapes, kiwis, apples, pears, oranges, mangos, and persimmons vinegars) composition. The strongest inhibitory effect on the formation of fluorescent AGEs was exhibited by the orange vinegar; a finding which was also correlated to the relatively high level of total flavonoids and total phenols content as well as to better effect on antioxidant activity. Then orange vinegar and its main polyphenol components were used to reveal the protective effect on cell damage induced by AGEs, which found that catechin had the best effect on cell protection. It was attributed to orange vinegar and its main polyphenol components to decreasing the expression levels of RAGE, which can active NADPH oxidase and lead to the increase of intracellular ROS. What’s more, they also inhibited the overexpression of p38MAPK, FOXO1, Bax and other inflammatory factors, which protected the cells from apoptosis (Fig. 4). Our research may contribute to the further development and development of fruit vinegar research and provide new ideas for subsequent research.

## CRediT authorship contribution statement

**Qian Wu:** Conceptualization, Methodology, Writing – review & editing. **Yingfei Kong:** Conceptualization, Data curation, Writing – review & editing. **Yinggang Liang:** Data curation, Methodology. **Mengyao Niu:** Investigation. **Nianjie Feng:** Conceptualization, Methodology, Supervision, Funding acquisition. **Chan Zhang:** Visualization. **Yonggang Qi:** Writing – review & editing. **Zhiqiang Guo:** Methodology, Data curation. **Juan Xiao:** Methodology. **Mengzhou Zhou:** Supervision. **Yi He:** Conceptualization, Supervision. **Chao Wang:** Funding acquisition.

## Declaration of Competing Interest

The authors declare that they have no known competing financial interests or personal relationships that could have appeared to influence the work reported in this paper.

## Data Availability

Data will be made available on request.
