# Supplementary material for: Protective mechanism of fruit vinegar polyphenols against AGEs-induced Caco-2 cell damage
Source: Food Chem X. 2023 Jun 5;19:100736. doi: 10.1016/j.fochx.2023.100736 (PMC10319990; doi:10.1016/j.fochx.2023.100736)
Supplement: Supplementary data 1 [file mmc1.docx]

**Supporting Information**

**Protective mechanism of fruit vinegar polyphenols against AGEs-induced Caco-2 cell damage**

Qian Wu ^a#^, Yingfei Kong ^a#^, Yinggang Liang ^a^, Mengyao Niu ^a^, Nianjie Feng ^a^*, Chan Zhang ^b^, Yonggang Qi ^a^, Zhiqiang Guo ^c^, Juan Xiao ^c^, Mengzhou Zhou ^a^, Yi He ^d^*, Chao Wang ^a^*

a Key Laboratory of Fermentation Engineering (Ministry of Education), Hubei Key Laboratory of Industrial Microbiology, National “111” Center for Cellular Regulation and Molecular Pharmaceutics, Hubei Research Center of Food Fermentation Engineering and Technology, Hubei University of Technology, Wuhan, 430068, Hubei, P.R. China

b Beijing Laboratory of Food Quality and Safety, School of Food and Chemical Engineering, Beijing Technology and Business University, Beijing 100048, China

c State Key Laboratory of Marine Resource Utilization in South China Sea/Ministry of Education, Key Laboratory of Food Nutrition and Functional Food of Hainan Province/Engineering Research Center of Utilization of Tropical Polysaccharide Resources/ School of Food Science and Engineering, Hainan University, Haikou, China

d National R&D Center for Se-rich Agricultural Products Processing, Hubei Engineering Research Center for Deep Processing of Green Se-rich Agricultural Products, School of Modern Industry for Selenium Science and Engineering, Wuhan Polytechnic University, Wuhan 430023, P. R. China

Email addresses: Qian Wu (wuqian@hbut.edu.cn), Yingfei Kong (kongyingfei0923@outlook.com), Yinggang Liang (Liangyinggang2021@163.com), Mengyao Niu (nmy18239532535@163.com), Chan Zhang ([zhangchan@th.btbu.edu.cn](mailto:zhangchan@th.btbu.edu.cn)), Yonggang Qi (qjg7520@163.com), Zhiqiang Guo (guozq@hainanu.edu.cn), Juan Xiao (xiaojuan209218@163.com), Mengzhou Zhou (zmzkelvin@163.com)

Corresponding author email addresses: Nianjie Feng ([njfeng@hbut.edu.cn](mailto:njfeng@hbut.edu.cn)), Yi He ([yi.he@whpu.edu.cn](mailto:yi.he@whpu.edu.cn)), Chao Wang ([wangchaohugong@sina.com](mailto:wangchaohugong@sina.com)), Tel numbers: +86-13720167990

^#^ indicates equal contribution.

**Contents:**

**Supporting Information Figure S1.** The content of total flavonoids and total phenols in different fruit vinegar. A, the content of total flavonoids; B, the content of total phenols. Different letters indicated significant differences (*P < 0.05*).

**Supporting Information Figure S2.** Antioxidant ability of different fruit vinegar. Each group has 3 similarities, and different letters indicate significant differences (*p<0.05*).

**Supporting Information Figure S3.** Relative content of various substances in fruit vinegar.

**Supporting Information Figure S4.** Analysis of fruit vinegar by liquid chromatography mass spectrometry.

**Supporting Information Figure S5.** The effect of fruit vinegar on the formation of AGEs. Different letters indicated significant differences (*P < 0.05*).

**Supporting Information Figure S6.** MTT results of orange vinegar and its main components. A, the cell viability of CML, OV diluted (orange vinegar diluted), CC (catechin), EC (epicatechin) and PA (*p*-coumaric acid); B, the cell viability of CML, CML+OV (orange vinegar), CML+CC (catechin), CML+EC (epicatechin) and CML+PA (*p*-coumaric acid). Different letters indicated significant differences (*P < 0.05*).

**Supporting Information Figure S7.** Effects of orange vinegar and its main components on the expression of inflammatory cytokines. A, the expression of TNF-α; B, the expression of IL-6. Different letters indicated significant differences (*P < 0.05*).

**
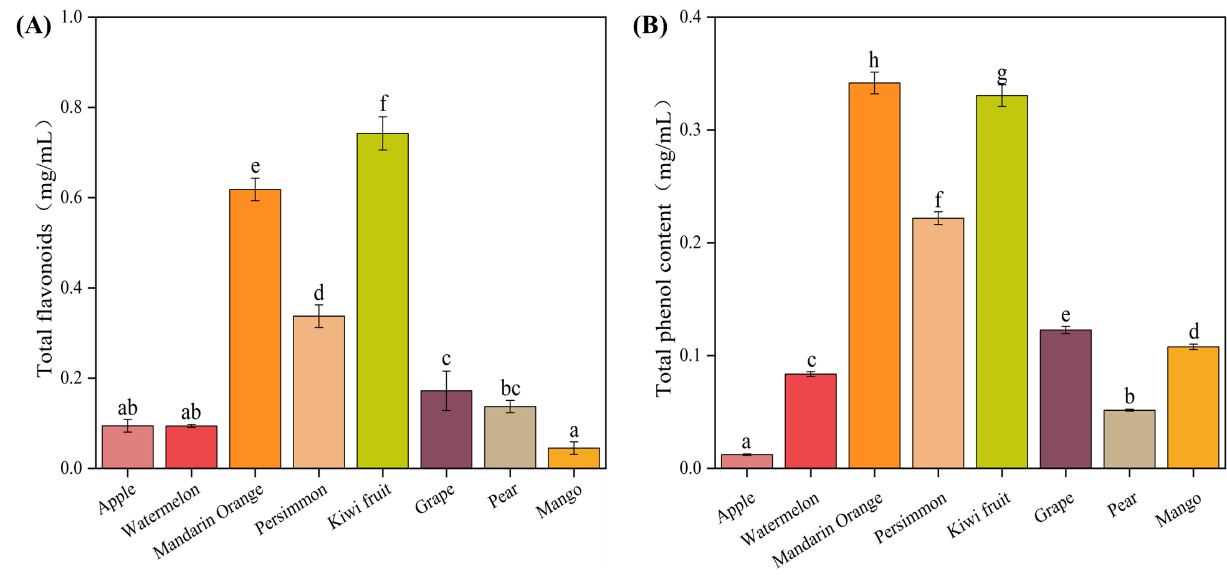
**

**Supporting Information Figure S1.**

**(A)**


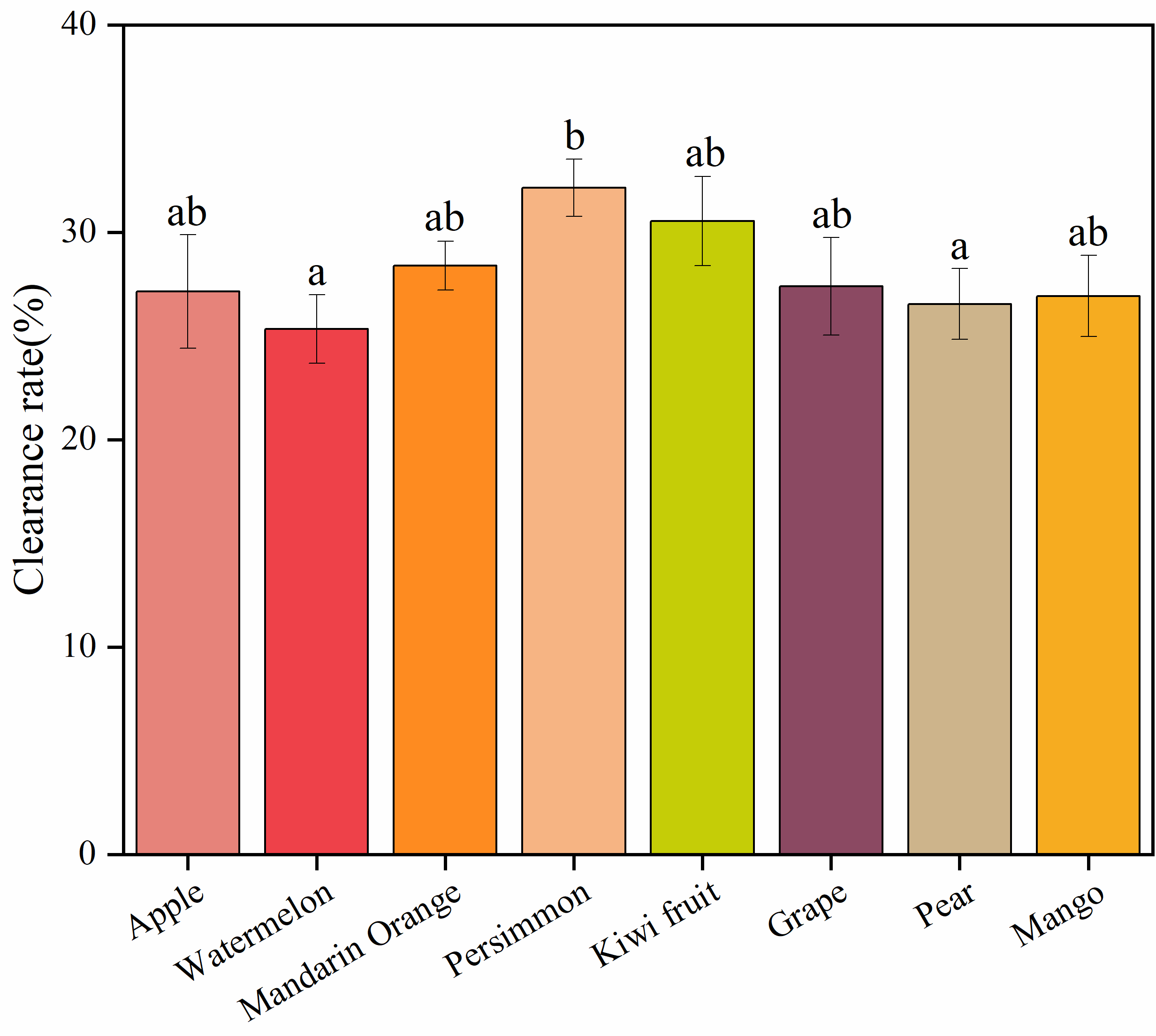


DPPH

**(B)**


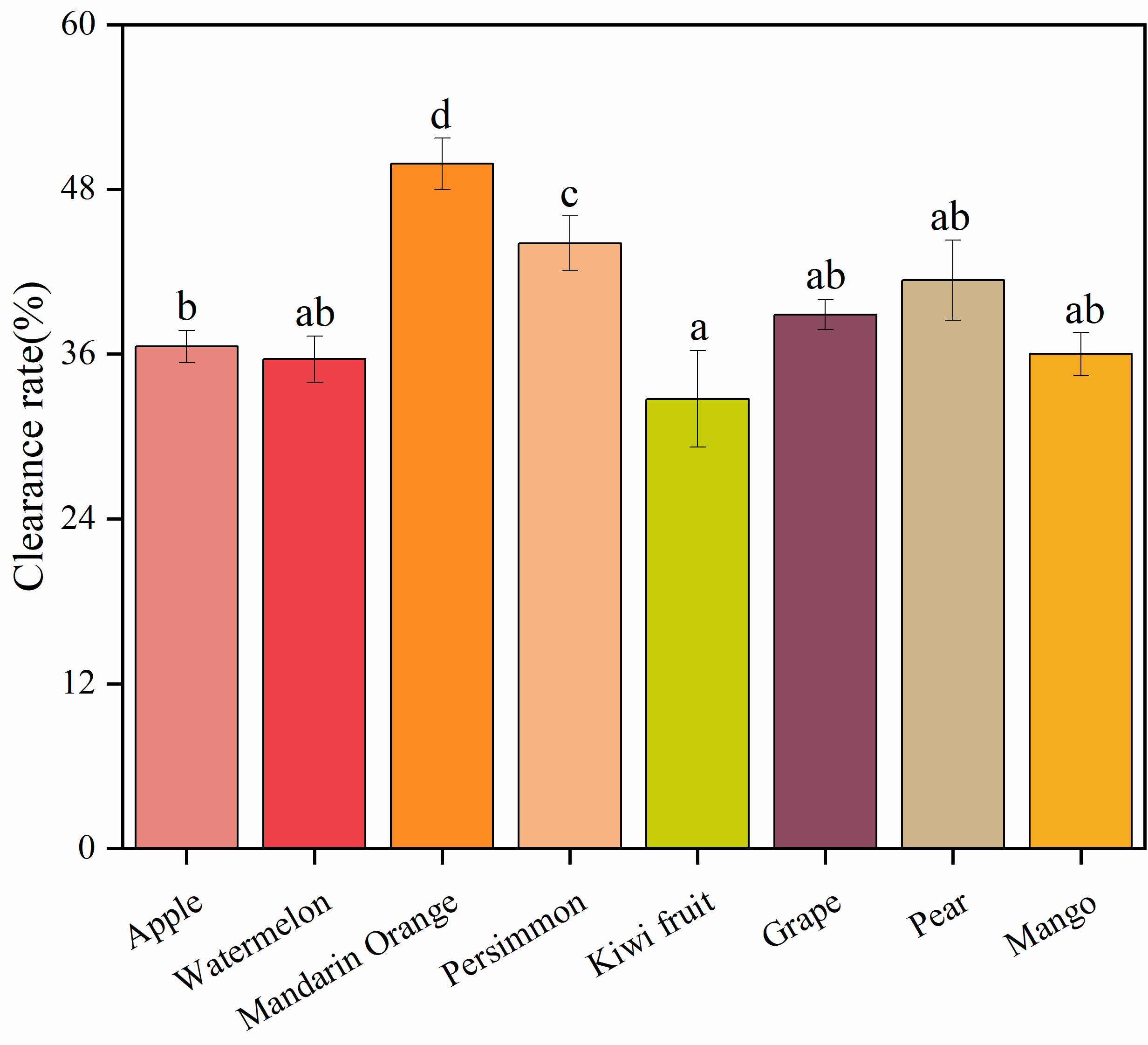


FRAP

**(C)**


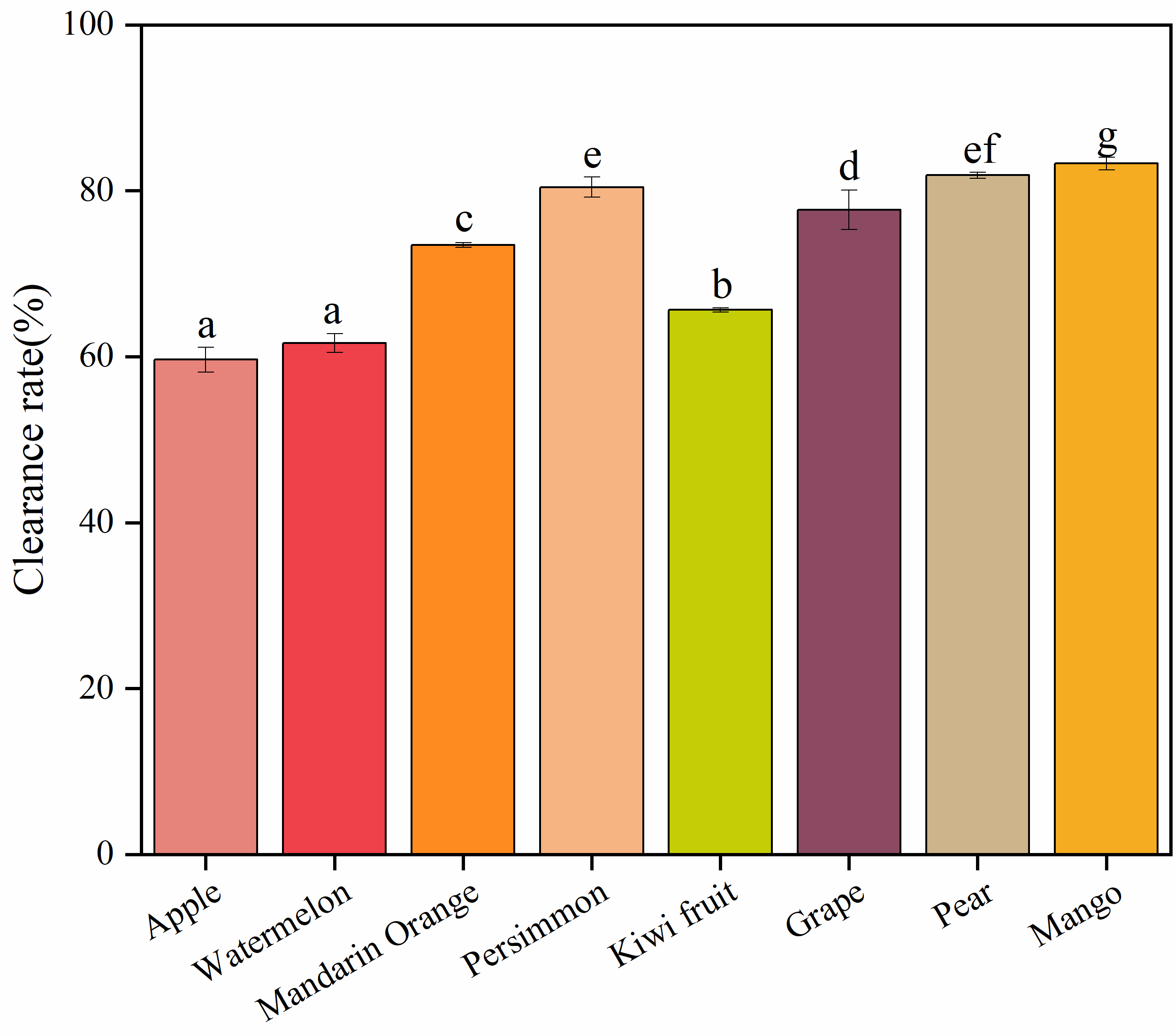


ABTS

**Supporting Information Figure S2.**

**Supporting Information Figure S3.**


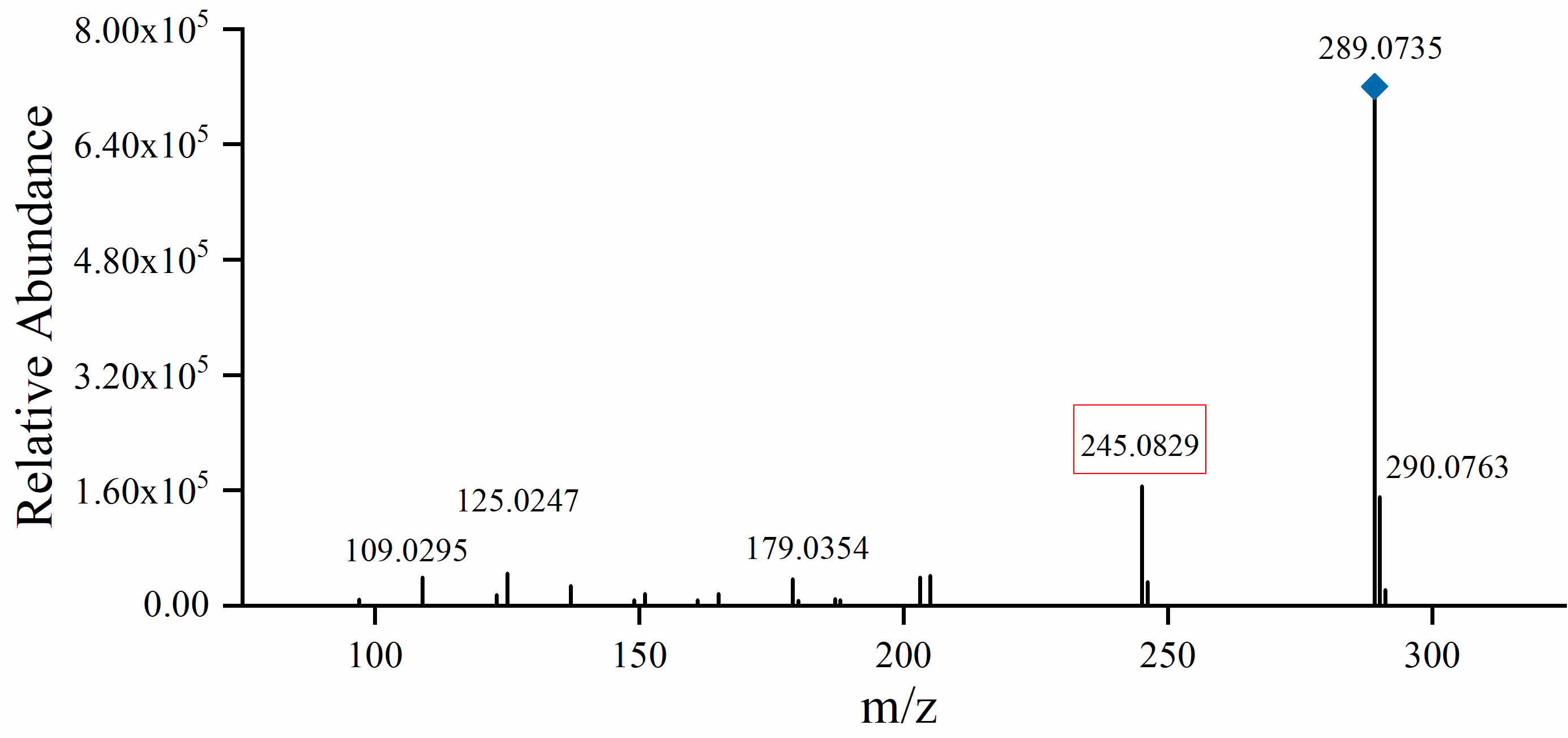


Catechin


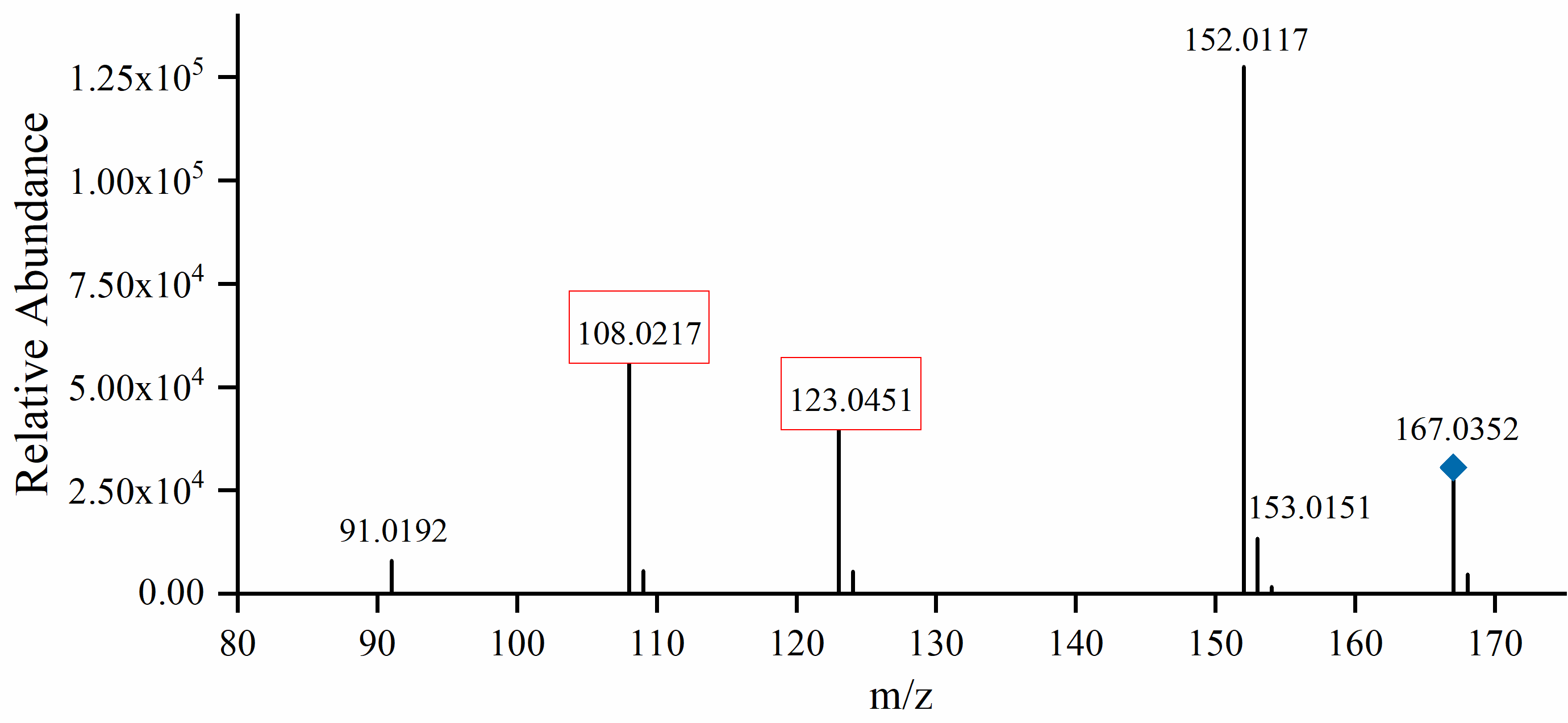


Vanillic acid


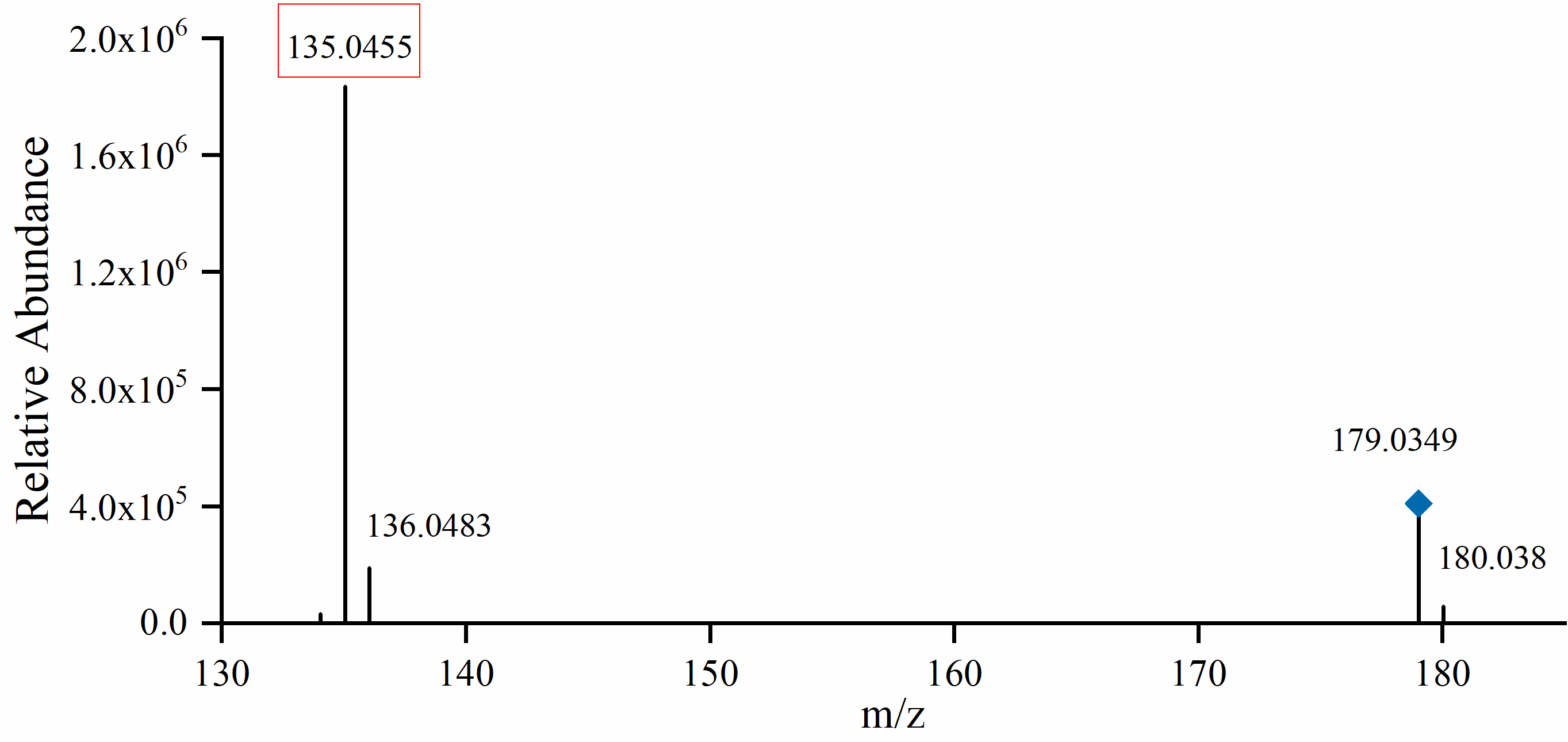


Caffeic acid


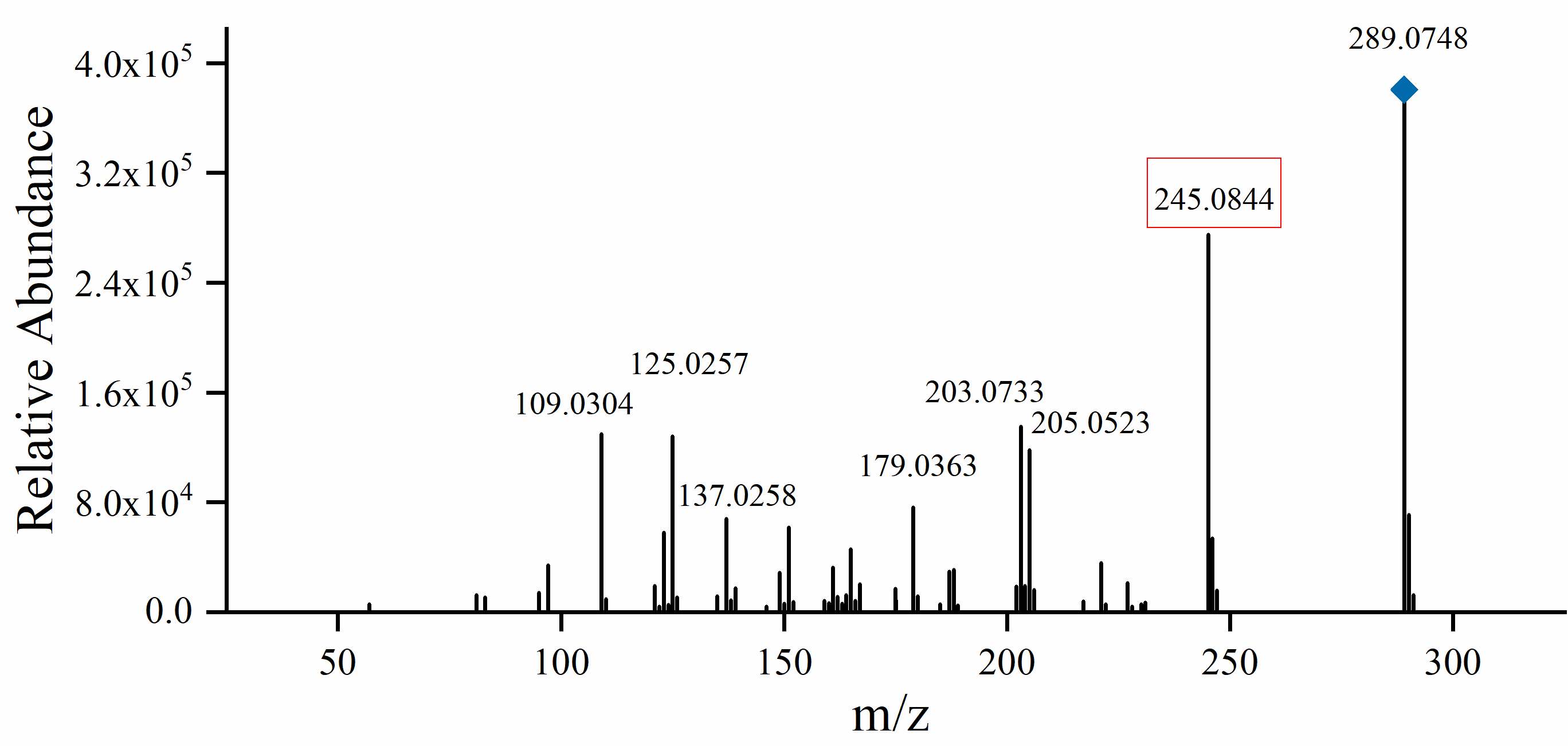


Epicatechin


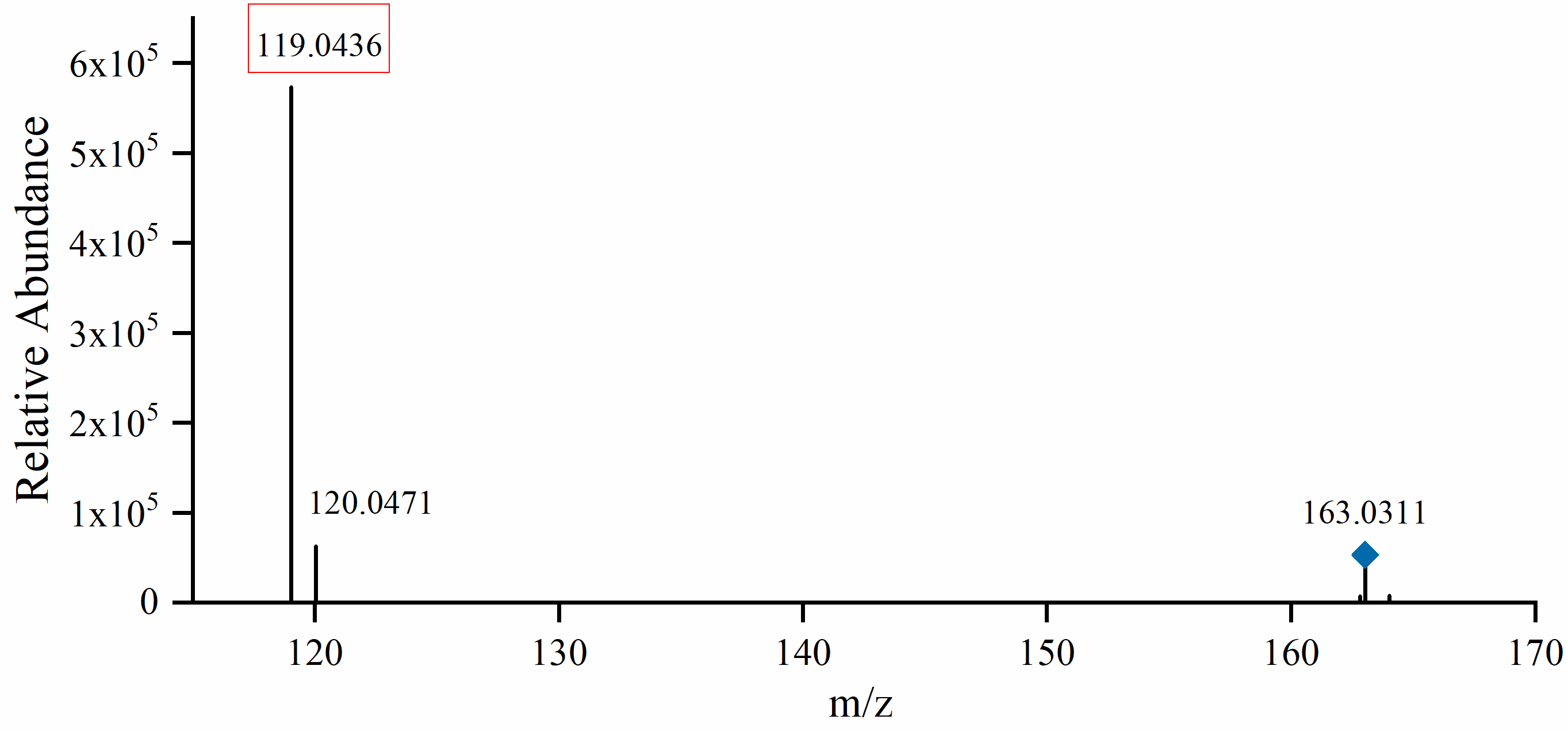


P-coumaric acid


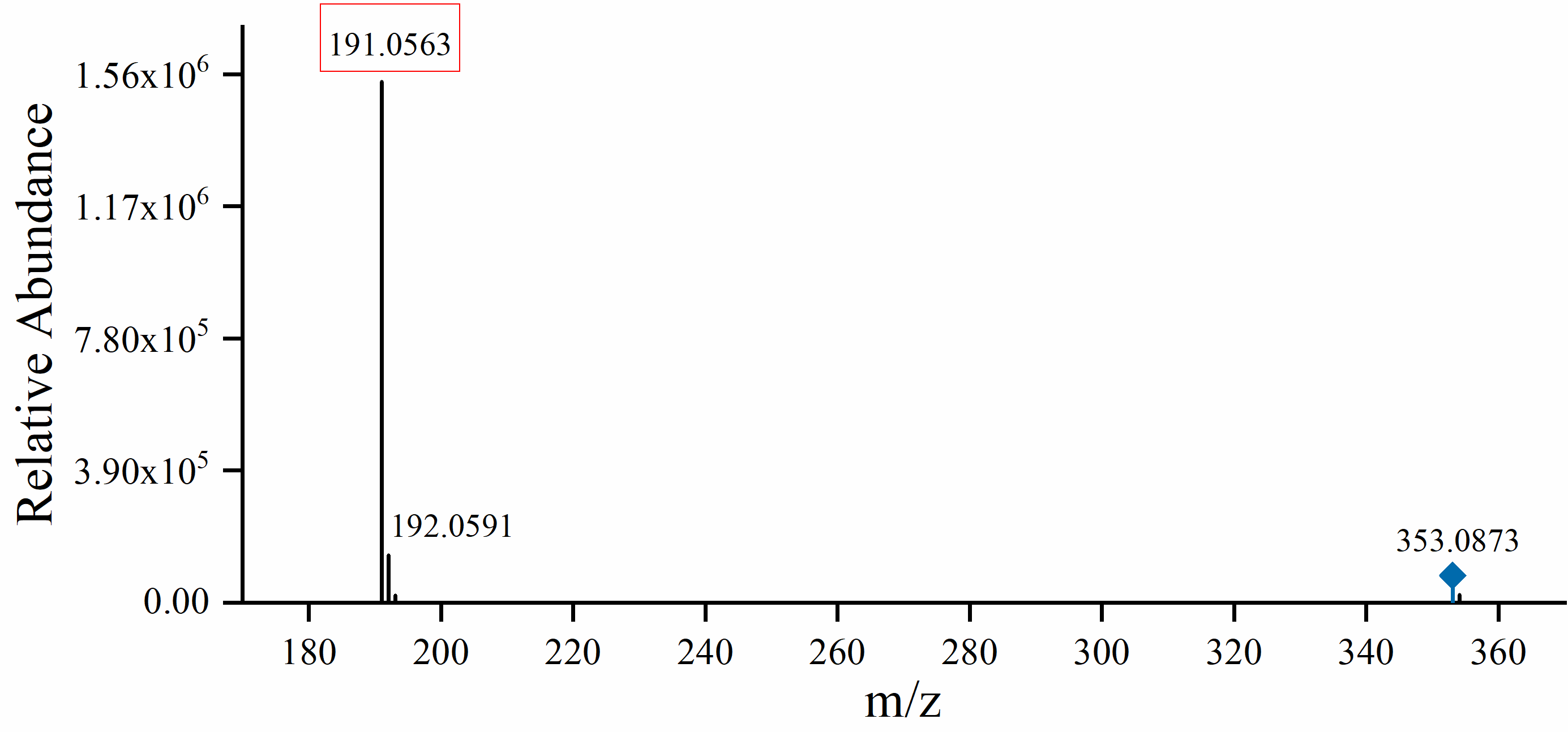


Chlorogenic acid


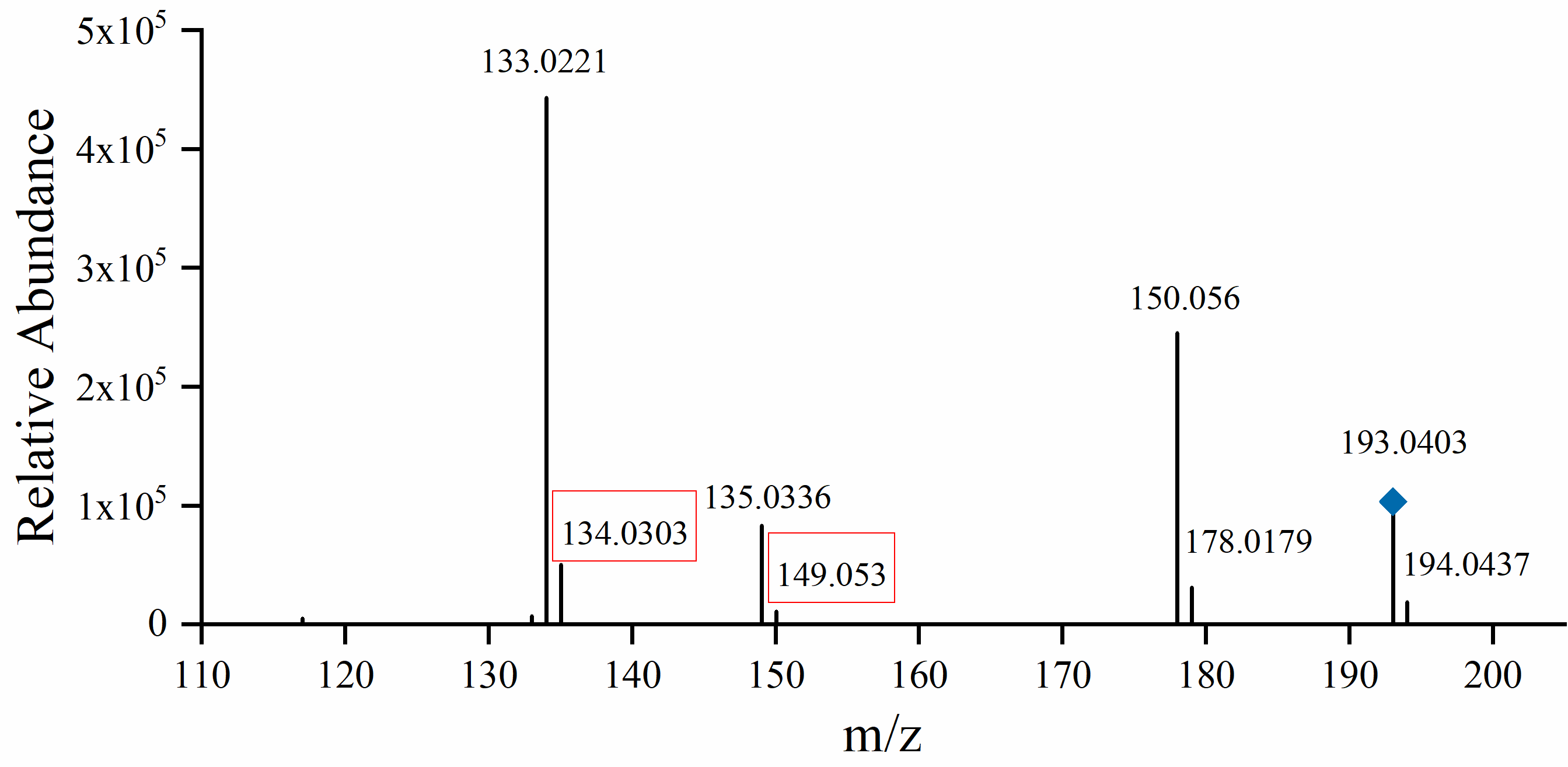


Ferulic acid

**Supporting Information Figure S4.**

**Supporting Information Figure S5**.

**(A)**

**(B)**

**Supporting Information Figure S6**.

**(A)**





**(B)**





**Supporting Information Figure S7**.
